# Supplementary material for: Depression, anxiety, stress and their associated factors among Ethiopian University students during an early stage of COVID-19 pandemic: An online-based cross-sectional survey
Source: PLoS One. 2021 May 28;16(5):e0251670. doi: 10.1371/journal.pone.0251670 (PMC8162693; doi:10.1371/journal.pone.0251670)
Supplement: Attachment — Submitted filename: Response to reviewers.docx [file pone.0251670.s004.docx]

**Point by point response for editor/reviewers comments**

# Manuscript title: Depression, anxiety, stress and their associated factors among Ethiopian University students during early stage of COVID-19 pandemic: An online based cross-sectional survey

**Manuscript ID**: PONE-D-20-39731

**Dear editor/reviewers**: Thank you for giving us the chance to revise the manuscript.

We have addressed all of the concerns raised. These modifications are also incorporated in the revised manuscript.

A. **Response to editor comments**
1. Please ensure that your manuscript meets PLOS ONE's style requirements, including those for file naming.

**Response**: Thank you. We have checked the manuscript for the journal requirements and made all necessary amendments in the revised manuscript.

2. Please clarify in the Methods section the criterion for participants to be social media users, and why this was in place.

**Response**: We prefer to use social media users because it enables us to collect the data without direct contact with the study participants, which is important to reduce the rate of spread of the COVID 19 pandemic. Based on your suggestion, we have also included this statement in method section of the revised manuscript (line 109 and 110).

3. Thank you for stating the following financial disclosure: "The funders had no role in study design, data collection and analysis, decision to publish, or preparation of the manuscript." At this time, please address the following queries:
a. Please clarify the sources of funding (financial or material support) for your study. List the grants or organizations that supported your study, including funding received from your institution.
b. State what role the funders took in the study. If the funders had no role in your study, please state: “The funders had no role in study design, data collection and analysis, decision to publish, or preparation of the manuscript.”
c. If any authors received a salary from any of your funders, please state which authors and which funders.
d. If you did not receive any funding for this study, please state: “The authors received no specific funding for this work.”
e. Please include your amended statements within your cover letter; we will change the online submission form on your behalf.

**Response: Thank you.** The authors received no specific funding for this work. We have also included this statement (see line 319).

4. In your Data Availability statement, you have not specified where the minimal data set underlying the results described in your manuscript can be found

**Response:** Thank you. We have modified the data availability statement based on your suggestion.

B. **Response to comments of reviewer 1**
1. Aside from minor language correction, I suggest including a similar study in the Middle East, whose findings echo this study:Alfawaz HA, Wani K, Aljumah AA, Aldisi D, Ansari MGA, Yakout SM, Sabico S, Al-Daghri NM. Psychological well-being during COVID-19 lockdown: Insights from a Saudi State University's Academic Community. J King Saud Univ Sci. 2021 Jan;33(1):101262.

**Response**: we have tried to revise and correct language errors throughout the paper. We have cited the paper you suggested in the revised paper as well (line 74 and 75).

2. I would also recommend the use of a flowchart to give a better picture of the respondents in the survey.

**Response**: Thank you very much for this suggestion. However, we believe that adding flow chart will increase the volume as we have used five tables and the use of flow chart will not add to much information as we believe.
