# Supplementary material for: Depression, anxiety, stress and their associated factors among Ethiopian University students during an early stage of COVID-19 pandemic: An online-based cross-sectional survey
Source: PLoS One. 2021 May 28;16(5):e0251670. doi: 10.1371/journal.pone.0251670 (PMC8162693; doi:10.1371/journal.pone.0251670)
Supplement: Attachment — Submitted filename: Response to editor.docx [file pone.0251670.s005.docx]

Response to editor’s comments (PONE-D-20-39731R1)

Dear editor, thank you very much for your comments regarding our manuscript.

Based on your request we have included the study participants’ flowchart and revised the manuscript for the language related issues thoroughly.

We have enclosed herewith the manuscript clean and track change versions along with the flow chart Tif format.

Thank you!
